# Supplementary material for: An encryption–decryption framework to validating single-particle imaging
Source: Sci Rep. 2021 Jan 13;11:971. doi: 10.1038/s41598-020-79589-0 (PMC7806625; doi:10.1038/s41598-020-79589-0)
Supplement: Supplementary file 1 — Supplementary Information. [file 41598_2020_79589_MOESM1_ESM.pdf]

An encryption-decryption framework to validating single-particle imaging: Supplementary Information

Zhou Shen[a,b], Colin Zhi Wei Teo[a,b], Kartik Ayyer[c,d], and N. Duane Loh[a,b,e]

[a]Centre for Bio-imaging Sciences, National University of Singapore, 14 Science Drive 4, 117557, Singapore
[b]Department of Physics, National University of Singapore, 2 Science Drive 3, 117551, Singapore
[c]Max Planck Institute for the Structure and Dynamics of Matter, Luruper Chaussee 149, 22761 Hamburg, Germany
[d]Center for Free-Electron Laser Science, Luruper Chaussee 149, 22761 Hamburg, Germany
[e]Department of Biological Sciences, National University of Singapore, 14 Science Drive 4, 117557, Singapore

Table 1: Multiplication table of basis unit quaternions

	$\mathbf{1}$	$\boldsymbol{i}$	$\boldsymbol{j}$	$\boldsymbol{k}$
$\mathbf{1}$	$\mathbf{1}$	$\boldsymbol{i}$	$\boldsymbol{j}$	$\boldsymbol{k}$
$\boldsymbol{i}$	$\boldsymbol{i}$	$-\mathbf{1}$	$\boldsymbol{k}$	$-\boldsymbol{j}$
$\boldsymbol{j}$	$\boldsymbol{j}$	$-\boldsymbol{k}$	$-\mathbf{1}$	$\boldsymbol{i}$
$\boldsymbol{k}$	$\boldsymbol{k}$	$\boldsymbol{j}$	$-\boldsymbol{i}$	$-\mathbf{1}$

1 Representing spatial rotation with quaternions

In this section, a brief introduction is given about the unit quaternion representation of rotation, which commonly occurs in computational geometry. Quaternions are points in a 4D real space (Q_0, Q_1, Q_2, Q_3). And the unit quaternion $(\sum_{i=0}^{3} Q_i^2 = 1)$ representation of a rotation has the following relation with the angle-axis pair representation, $(\theta \in [0, 2\pi), \hat{\boldsymbol{n}})$,

$$Q = (\cos\frac{\theta}{2}, \sin\frac{\theta}{2} \cdot \hat{\boldsymbol{n}}), \tag{1}$$

where $\hat{\boldsymbol{n}}$ is the axis of the rotation and θ is the rotation angle.

The combination of two rotations is mapped to a special multiplication, which makes quaternions into an algebra. To define that multiplication, it is easier to rewrite Eqn. (1) into $Q = Q_0\mathbf{1} + Q_1\boldsymbol{i} + Q_2\boldsymbol{j} + Q_3\boldsymbol{k}$, where $\mathbf{1}$, $\boldsymbol{i}$, $\boldsymbol{j}$, $\boldsymbol{k}$ are four basis unit quaternions $(1, 0, 0, 0)$, $(0, 1, 0, 0)$, $(0, 0, 1, 0)$, $(0, 0, 0, 1)$. The multiplication between any two quaternions can be extended from the multiplication table (Table 1) of these four bases.

Simply applying this bases multiplication shows that the conjugate, $Q^* \equiv Q_0\mathbf{1} - Q_1\boldsymbol{i} - Q_2\boldsymbol{j} - Q_3\boldsymbol{k}$, of a unit quaternion Q is its inverse, $QQ^* = \mathbf{1}$. This conclusion also can be verified by considering the quaternion representation of a rotation $(\theta, \hat{\boldsymbol{n}})$ and its inverse $(\theta, -\hat{\boldsymbol{n}})$. Another helpful corollary derived from the multiplication is about calculating the natural geodesic distance between two rotations, $\Omega_A, \Omega_B \in \mathrm{SO}(3)$. This distance is defined as the angle of rotation, θ, of the joint rotation operation $\Omega_A \cdot \Omega_B^{-1}$, or $\theta(Q_A, Q_B) = 2 \cdot \arccos \sum_{i=0}^{3} Q_{Ai} Q_{Bi}$ in the quaternion representation.

It should be noted that the positions of the OPD clusters in Fig. 9 (main text) are centro-symmetric. The reason is that rotating an object by θ along axis $\hat{\boldsymbol{n}}$, could also be expressed as rotating it by $2\pi - \theta$ along axis $-\hat{\boldsymbol{n}}$. However, the quaternions representations of these two equivalent rotations, $(\theta, \hat{\boldsymbol{n}}) \to Q$ and $(2\pi - \theta, -\hat{\boldsymbol{n}}) \to -Q$, are different by Eqn. (1). Hence, we call the unit quaternion representation a *double cover* of SO(3) group (also known as the 3D rotation group).
